# The large plasmid carried class 1 integrons mediated multidrug resistance of foodborne *Salmonella* Indiana

**DOI:** 10.3389/fmicb.2022.991326

**Published:** 2022-10-14

**Authors:** Xuefeng Wang, Tian Wang, Mengjiao Guo, Chengcheng Zhang, Zongyi Bo, Yantao Wu, Guoxiang Chao

**Affiliations:** ^1^College of Veterinary Medicine, Yangzhou University, Yangzhou, China; ^2^College of Medicine, Yangzhou University, Yangzhou, China

**Keywords:** *Salmonella* Indiana, class 1 integrons, integrase, conjugation, multidrug resistance

## Abstract

*Salmonella enterica* serovar Indiana (*S*. Indiana) has aroused widespread concern as an important zoonotic pathogen. The molecular mechanism of multidrug resistance (MDR) in *S*. Indiana is not known and should be assessed. We aim to investigate the molecular mechanism of MDR and the importance of large plasmids carried class 1 integrons in the MDR of foodborne *S*. Indiana. Class 1 integrons in 48 *S*. Indiana isolates and 200 isolates of 7 other *Salmonella* serotypes were detected by polymerase chain reaction (PCR). To analyze the antimicrobial resistance genes (ARGs) of two *S*. Indiana isolates, designated *S*. Indiana 15 and *S*. Indiana 222, next-generation sequencing (NGS) was performed, and the resulting sequences were compared with the complete nucleotide sequences of *S*. Indiana D90 and *S*. Indiana C629. Comparative functional analysis was conducted between the *intI1* (1,014 bp) of *S*. Indiana 222 and the *intI1* (699 bp) of *S*. Indiana 15. Plasmid conjugation transfer analysis was performed to analyze the horizontal gene transfer of the integrons-related resistance genes with integron-positive and integron-negative *Salmonella* isolates. 64.58% of *S*. Indiana isolates carried class 1 integrons, which was significantly higher than that of other *Salmonella* serotypes (*p* < 0.001). The NGS results showed that the *S*. Indiana 15 and *S*. Indiana 222 isolates carried a large plasmid with a class 1 integron and multiple ARGs, similar to *S*. Indiana D90 and *S*. Indiana C629. Two integrases found in *S*. Indiana isolates belong to class 1 integrases and could integrate resistance genes into specific integration sites of the integrons. The conjugation frequency of *intI1* (1,014 bp) was 6.08 × 10^−5^, which was significantly higher than that of *intI1* (699 bp) (*p* < 0.01). The large plasmids carrying a class 1 integron and the number of ARGs were strongly correlated (*p* < 0.001). The conjugation frequency of integron-positive *S*. Indiana recipient isolates was significantly higher than that of integron-negative recipient isolates (*p* < 0.05). *S*. Indiana containing large plasmids carrying a class 1 integron more easily captured resistance genes from other bacteria (*S*. Enteritidis and *S*. Derby), which could be an important cause of the emerging pandemic of MDR clones.

**Graphical abstract:**
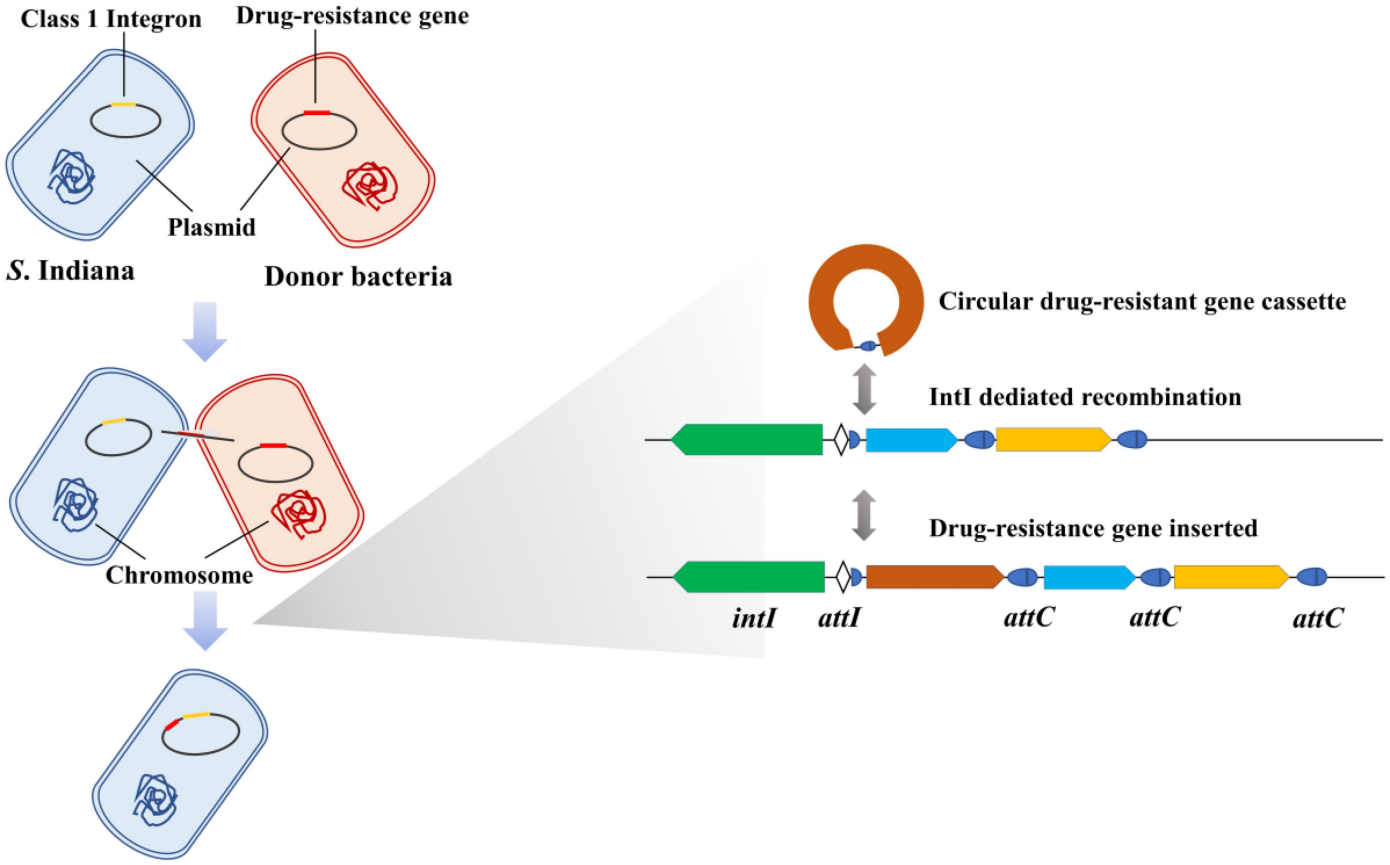
*S*. Indiana containing large plasmids carrying a class 1 integron more easily captured resistance genes from other bacteria (*S*. Enteritidis and *S*. Derby), which could be an important cause of the emerging pandemic of MDR clones.

*S*. Indiana containing large plasmids carrying a class 1 integron more easily captured resistance genes from other bacteria (*S*. Enteritidis and *S*. Derby), which could be an important cause of the emerging pandemic of MDR clones.

## Introduction

Throughout the world, foodborne pathogens are still causing many intestinal diseases in humans, resulting in substantial health and economic burdens. The majority of the reported foodborne illness outbreaks are caused by known pathogens such as Norovirus, Rotavirus, Campylobacter, *Salmonella*, Hepatitis A virus (HAV), *Staphylococcus aureus* (*Staph*), *Listeria monocytogenes*, and Shiga toxin-producing *Escherichia coli* (Mozaffari Nejad et al., 2014; Hamzavi et al., 2018). *Salmonella enterica*, as an important zoonotic pathogen in China, not only causes massive economic losses to the livestock and poultry industries, but also poses a threat to human health (CDC, 2017, 2018; Hu et al., 2017). In recent years, salmonellosis caused by *S*. Indiana (ST17) clone has been increasing (Bai et al., 2015; CDC, 2018). In our previous research, nearly all of the *S*. Indiana isolates were isolated from chicken industry chains, and human *Salmonella* infections could result from transmission *via* poultry products (Chao et al., 2017). A previous study showed that *S*. Indiana had low pathogenic than other serotypes of *Salmonella* (Wang et al., 2020), and carried a large number of antimicrobial resistance-encoding genes, which made *S*. Indiana a repository of drug resistance genes (Michael and Schwarz, 2016; Chao et al., 2017; Wang J. et al., 2017).

Integrons were genetic elements first found in multidrug-resistant bacteria (Stokes and Hall, 1989), which play an important role in the transmission of drug resistance genes (Ng et al., 1999; Foley et al., 2013; Bakkeren et al., 2019). Research showed that 9% of either partially or completely sequenced bacterial genomes harbored integrons (Boucher et al., 2007). The class 1 integrons are widespread in Gram-negative bacterial pathogens and are the most well-known integrons (Hall et al., 1999; Clark et al., 2000; White et al., 2001). Integrons are characterized by their capability to capture and delete gene cassettes through site-specific recombination mediated by integron integrase (Collis and Hall, 1992a,b; Collis et al., 1993). The DNA integrase is encoded by the *int* gene, which is located in the 5′ conserved segment (Hall and Vockler, 1987; Stokes and Hall, 1989). A shorter core recombination site, defined as the consensus GTTRRRY (R, purine; Y, pyrimidine), was predicted from comparisons of sequences at the boundaries of con-served and insert regions and at the boundaries between gene pairs (Stokes and Hall, 1989; Hall et al., 2010). Core recombination sites are found at the junction of the 5′ conserved segment and the first insert gene and as the last 7 bases of 59-base elements (Fluit and Schmitz, 1999).

As described herein, we studied the presence of class 1 integrons in *S*. Indiana isolates and in 7 other serotypes of *Salmonella* isolates and ascertained the relationship between class 1 integrons and the multidrug resistance of *S*. Indiana isolates, to reveal the causes of the multidrug resistance of *S*. Indiana. We also determined the complete nucleotide sequences of two *S*. Indiana isolates, designated *S*. Indiana 15 and *S*. Indiana 222, which were compared with the complete nucleotide sequences of two *Salmonella* strains (*S*. Indiana D90 and *S*. Indiana C629) found in the NCBI database. We compared the differences of the two integrase gene sequences, which were found from *S*. Indiana 15 and *S*. Indiana 222, and determine the integration frequency of these two integrases. The plasmid conjugation transfer assay was performed to compare the conjugation frequencies of integron-positive *S*. Indiana recipient isolates and integron-negative recipient isolates.

The goal of this study was to explore the molecular mechanism of multidrug resistance in *S*. Indiana and the importance of large plasmids carried class 1 integrons in the MDR of foodborne *S*. Indiana. Our findings contribute to the vital work of the causes of multidrug resistance at the genetic level.

## Materials and methods

### Isolate collection, plasmids, and media

All isolates used in our study, including 48 *S*. *Indiana* isolates and 200 isolates of 7 other *Salmonella* serotypes, *S*. *Enteritidis* (*n* = 94), *S*. *Derby* (*n* = 36), *S*. *London* (*n* = 10), *S*. *Senftenberg* (*n* = 10), *S*. *Typhimurium* (*n* = 30), *S*. *Thompson* (*n* = 9), and *S*. *Choleraesuis* (*n* = 11), were collected from the baseline survey of *Salmonella* in food industry supply chains in Jiangsu, China (Chao et al., 2017). All isolates in this study were collected from various sources of 7 cities in Jiangsu province, China. Of these, 65 and 31 were sourced from chicken farms and pig farms, respectively. Fifty were collected from a veterinary diagnostic laboratory of Yangzhou University, having been originally isolated from sporadic cases of diseased chickens at different, scattered, individual farms. Seven isolates were acquired from raw meat delivered to restaurants. Forty-four were taken from human carriers working in the food industry, and 51 were obtained from patients with food-related diarrhea at various hospitals. Two isolates with more resistance genes, designated *S*. Indiana 15 (accession number: CP092258-CP092263) and *S*. Indiana 222 (accession number: CP031189-CP031191), were used for NGS. According to the difference in the resistance genes carried by *Salmonella* and the existing antibiotics in our laboratory, seven isolates, designated *S*. Indiana 47, *S*. Indiana 89, *S*. Indiana 388, *S*. Derby 146, *S*. Indiana 368, *S*. Enteritidis 147, and *S*. Enteritidis 242, were used for the plasmid conjugation transfer assay. *Salmonella Indiana* D90 (accession number: CP022451) and *S*. *Indiana* C629 (accession number: CP015725) were found in the NCBI database for comparison with *S*. Indiana 15 and *S*. Indiana 222, because their serotypes were the same and they carried a large number of resistance genes, similar to *S*. Indiana 15 and *S*. Indiana 222. All isolates were confirmed by VITEK Gram-negative identification cards (bioMérieux Inc., Hazelwood, MO, United States), and serotyped according to the Kauffmann–White classification scheme. MLST was performed on all isolates. Seven housekeeping genes (*aroC*, *dnaN*, *hemD*, *hisD*, *purE*, *sucA*, and *thrA*) were sequenced to determine allelic profiles.

*Escherichia* coli BL21 (DE3) was obtained from TransGen Biotech Co., Ltd. Plasmid pUC19 was obtained from TransGen Biotech Co., Ltd., and plasmid pACYC184 was collected in the Animal Infectious Disease Laboratory School of Veterinary Medicines, Yangzhou University, Jiangsu.

The *E*. coli BL21 (DE3) strain was used for expression of integrase *intI1*. All bacterial strains were cultured at 37°C for propagation of the plasmid, on Luria-Bertani (LB) medium or LB agar supplemented, as necessary, with ampicillin (50 μg/ml), chloramphenicol (25 μg/ml), and trimethoprim (20 μg/ml).

### Detection of class 1 integrons

The class 1 integrase gene *intI1* was detected with two pairs of primers based on the sequence of class 1 integrons (Table 1). *S*. Indiana 222 was used as a positive control, which was confirmed by NGS to carry class 1 integrons, and ddH_2_O was used as a negative control. The sequences obtained were compared using the Integron Database INTEGRALL^1^ and the BLAST program.^2^

**Table 1:** Sequencing and PCR primers.

Primer	Sequence (5′-3′)	PCR product (bp)
intI1-F1	ATGAAAACCGCCACTGCG	1,014
intI1-R1	CTACCTCTCACTAGTGAGGGGCG	
intI1-15-F	CCGAGGATGCGAACCACTTC	373
intI1-15-R	CCGCCACTGCGCCGTTACCA	
intI1-F	GCGCTGCAGAATGAAAACCGCCACTGCG	1,033
intI1-R	GCGGAATTCCTACCTCTCACTAGTGAGGGGCG	
INT-F	GCATCGATGCTGTAAGCGAAGCGAACGA	2,330
INT-R	GCGGATATCCTACCTCTCACTAGTGAGGGGCG	
AAD-F	GCGAGTACTCTGCAAGGAGCCTTATTGTG	987
AAD-R	GCGCCATGGATCAGTGGCAAGAGGTTGG	
intI15-F	GCGCTCGAGCTATTTGCAACAGTGCCGC	717
intI15-R	GCCTGCAGAATGAAAACCGCCACTGCG	
INT15-F	GCGAGTACTCTATTTGCAACAGTGCCGC	1,526
INT15-R	GCGCCATGGAGCGTGGGACAGCTGCTT	
AAD15-F	GCATCGATTGCAAGGAGCCTTATTGTGC	985
AAD15-R	GCGGATATCATCAGTGGCAAGAGGTTGG	
5’CS	GGCATCCAAGCAGCAAGC	

### NGS

*Salmonella* Indiana 15 and *S*. Indiana 222 isolates were used for NGS and have been identified as MDR strains (Chao et al., 2017). These two strains were cultured on Lennox L Broth Base (LB; Invitrogen™ by Life Technologies, CA, United States) for 16–18 h. Genomic DNA was isolated from the two *Salmonella* isolates using a Bacteria DNA Kit (TAKARA). The extracted DNA was subjected to quality inspection by Invitrogen Qubit2.0 and 0.8% agarose gel electrophoresis. DNA qualified by quality inspection was subjected to subsequent sequencing. Paired-end libraries with insert sizes of approximately 400 bp were prepared by following Illumina’s standard genomic DNA library preparation procedure. Purified genomic DNA was sheared by Covaris into smaller fragments with a desired size. The qualified Illumina paired-end library was used for Illumina HiSeq sequencing (PE150 mode, Shanghai Biozeron Co., Ltd). After trimming based on base quality, which involved identifying contamination reads and correcting the PacBio long reads, we used ABySS^3^ to perform genome assembly with multiple *k*-mer parameters and ultimately pinpointed the optimal assembly. We next used Canu^4^ to assemble the PacBio-corrected long reads, and GapCloser software was subsequently applied to fill the remaining local inner gaps and correct the single-nucleotide polymorphism^5^ for the final assembly results.

We analyzed resistance genes using the Comprehensive Antibiotic Resistance Database (CARD)^6^ and identified features by referencing the ORF Finder program.^7^ The integrons were analyzed using the Integron Database INTEGRALL (see Footnote 1).

### Comparison of the integration efficiency of two class 1 integrases *intI1*

*Salmonella* Indiana strain 222 containing integron which sequence was the same as the integron in the plasmid pCTX-M3 (GenBank accession number: AF550415.2). Plasmids were extracted by the use of plasmid kits (Corning Life Sciences Co., Ltd) according to the recommendations of the manufacturer. Whole-cell DNA from bacteria used for the construction of recombinant plasmids was isolated by the use of TaKaRa MiniBEST Bacterial Genomic DNA Extraction Kit (TaKaRa Biotechnology Co., Ltd), following the operation manual. The primers used are listed in Table 1. The oligonucleotides were obtained as lyophilized purified products from GenScript Biotech Co., Ltd., Nanjing, China, and were diluted to working concentrations with deionized water. Final reaction mixtures consisted of 0.5 μl of DNA template, 10× buffer 5 μl, dNTPs 8 μl, 1 μl each of forward and reverse oligonucleotide primers, and 0.5 μl *TransTaq*® DNA Polymerase High Fidelity (GenScript Biotech Co., Ltd). The final volume was made up to 50 μl with water. After denaturation at 95°C for 5 min, 30 cycles, each consisting of 95°C for 1 min, 55°C for 1 min, and 72°C for 60 to 150 s (depending on the expected product size), were performed, followed by a final extension at 72°C for 5 min. The amplification products were detected by gel electrophoresis of 5 μl of the reaction mixture in agarose gels (1% molecular biology-grade agarose in 1 × TBE) containing ethidium bromide (4 μg/ml). The PCR products were purified on Axygen columns (Corning Life Sciences Co., Ltd). Then posted it to the General Biosystems Co., Ltd. for sequencing, and then proceed to the next experiment after confirming the sequence was correct.

**Figure 1:**
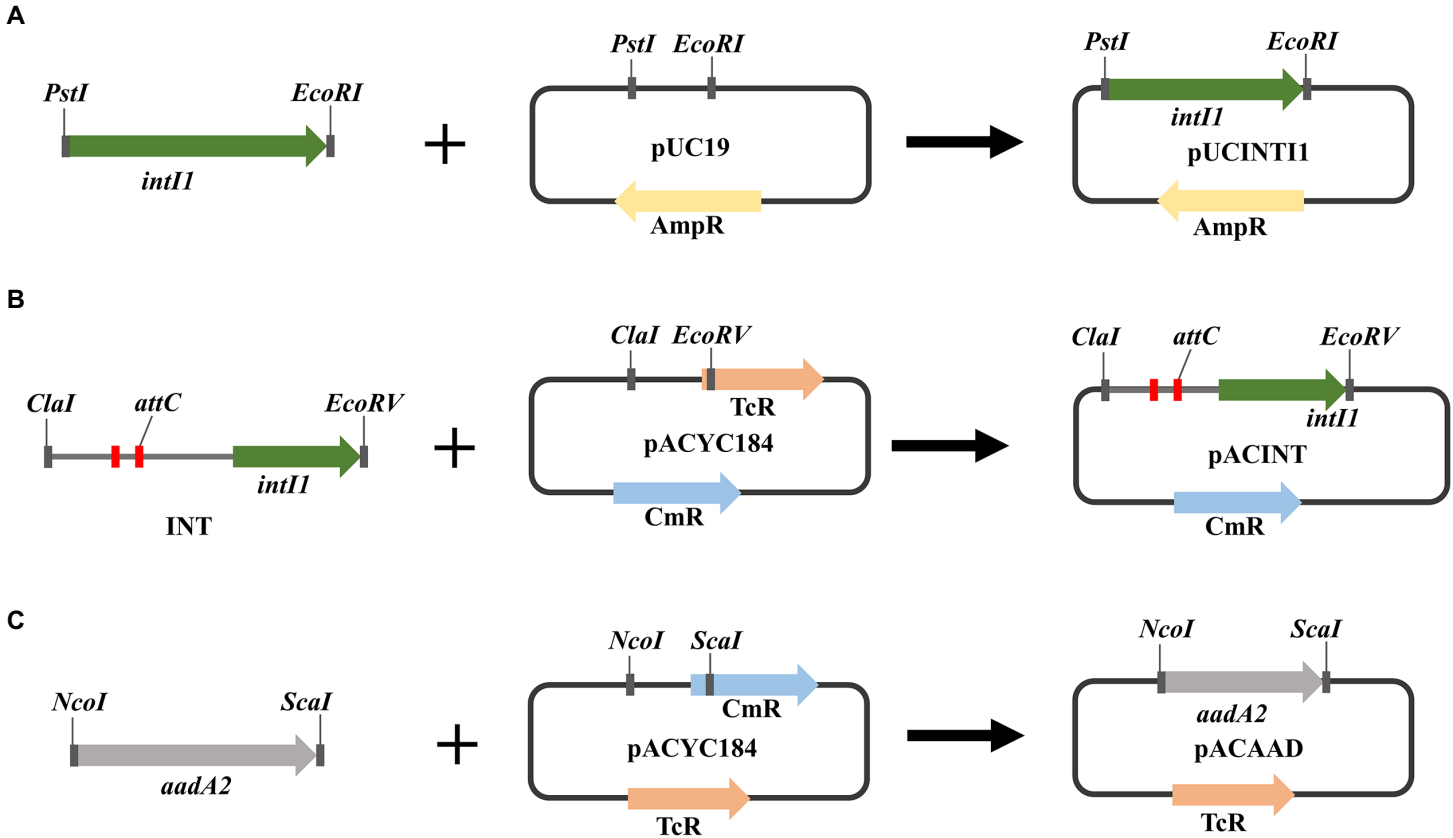
**(A)** Schematic diagram of recombinant plasmid pUCINTI1. **(B)** Schematic diagram of recombinant plasmid pACINT. **(C)** schematic diagram of recombinant plasmid pACAAD.

Recombinant plasmids were constructed as the following description (Figure 1). Restriction endonucleases and T4 DNA ligase were used according to the recommendations of the manufacturer (GenScript Biotech Co., Ltd). The integron integrase *intI1* gene was cloned into pUC19 between *EcoRI* and *PstI* restriction sites to obtain plasmid pUCINTI1. The integron gene and *aadA2* gene cassette were cloned into pACYC184 between *ScaI* and *NcoI*, *EcoRV* and *ClaI* restriction sites, respectively, to obtain plasmids pACINT and pACAAD. The resulting plasmids pUCINTI1, pACINT, and pACAAD were then transformed by heat shock into *E*. coli BL21(DE3) competent cells following the standard protocol.

*Escherichia* coli strain BL21(DE3) containing plasmids pUCINTI1, pACINT, and pACINAD was grown for 12 h in Luria-Bertani (LB) medium supplemented with Ampicillin, trimethoprim, IPTG (10 μg/ml), meanwhile *E*. coli strain BL21(DE3) containing plasmid pACINT was used as control. Taken an appropriate amount of bacterial solution for serial dilution and spread it on an LB agar plate supplemented with ampicillin (50 μg/ml) and chloramphenicol (25 μg/ml), and cultured overnight at 35°C. Then we counted the number of bacterial clones on the LB agar plate, and detected by polymerase chain reaction (PCR) to determine whether the *aadA2* gene was integrated. The conjugation frequency (Fc) is calculated according to the following formula: Fc = T/R (T represents the number of transconjugants colonies, R represents the number of all colonies).

### Plasmid conjugation transfer assay

Based on the results of class 1 integrons, integron-negative *S*. Enteritidis 147, *S*. Derby 368, and *S*. Enteritidis 242 were used as donor strains that provided drug resistance genes to the recipient strains. Integron-negative *S*. Indiana 388 and integron-positive *S*. Derby 146, *S*. Indiana 89, and *S*. Indiana 47 were used as recipient strains that accepted and integrated drug resistance genes. For the above 7 *Salmonella* strains, we identified the types of plasmids by using polymerase chain reaction (PCR)-based replicon typing (PBRT) (Carattoli et al., 2005; Carloni et al., 2017).

A plasmid conjugation transfer assay was performed by following a published method (Lomovskaya et al., 2017). The donor and recipient strains were processed by mixing 50 μl of overnight cultures grown in Lennox L Broth (LB) (OD_600_ = 0.5) in a sterile test tube and then spreading the mixture across polyvinylidene difluoride (PVDF) filtration membranes attached to LB agar plates. After the plates were incubated at 37°C overnight, the PVDF filtration membranes on the plates were removed by sterile forceps, and the confluent growth was suspended in 5 ml of LB. A tenfold dilution series (100 μl) of that suspension was next plated onto selective plates containing tetracycline (10 μg mL^−1^) and chloramphenicol (25 μg mL^−1^). After the plates were incubated at 37°C overnight, the number of transconjugant colonies was counted. Another tenfold dilution series (100 μl) of the suspension was plated onto selective plates containing chloramphenicol (25 μg mL^−1^) or tetracycline (10 μg mL^−1^) and incubated at 37°C overnight, and the number of recipient isolate colonies was counted. Once the experiment was performed in triplicate, the conjugation frequency (Fc) was calculated according to *Fc* = *T/R*, in which *T* represents the number of transconjugant colonies and *R* represents the number of recipient isolate colonies. Finally, the transconjugant colonies were tested by PCR to determine whether the resistance genes were transferred to the recipient isolates.

### Statistical analysis

All data were analyzed using Statistical Product and Service Solutions (SPSS) version 25.0 (IBM, Armonk, NY, United States). The correlations between the large plasmids carrying class 1 integrons and the number of antibiotic resistance genes were analyzed by calculating Pearson correlation coefficients. The independent samples *t*-test was used to compare the differences between the conjugation frequency (Fc) values, and a *p*-value less than 0.05 was considered to indicate statistical significance.

## Results

### Detection of class 1 integrons

Class 1 integrons accounted for 64.58% (31/48), 44.44% (4/9), 27.78% (10/36), 26.67% (8/30), and 1.06% (1/94) of the integrons in *S*. Indiana, *S*. Thompson, *S*. Derby, *S*. Typhimurium, and *S*. Enteritidis isolates, with an average of 23.39% (58/248) of the integrons of all the *Salmonella* isolates being Class 1 integrons. No class 1 integrons were detected in the *S*. Senftenberg or *S*. Choleraesuis isolates. The proportion of *S*. Indiana carrying class 1 integrons was significantly higher than that in the other *Salmonella* serotypes (*p* < 0.001).

### NGS

**Figure 2:**
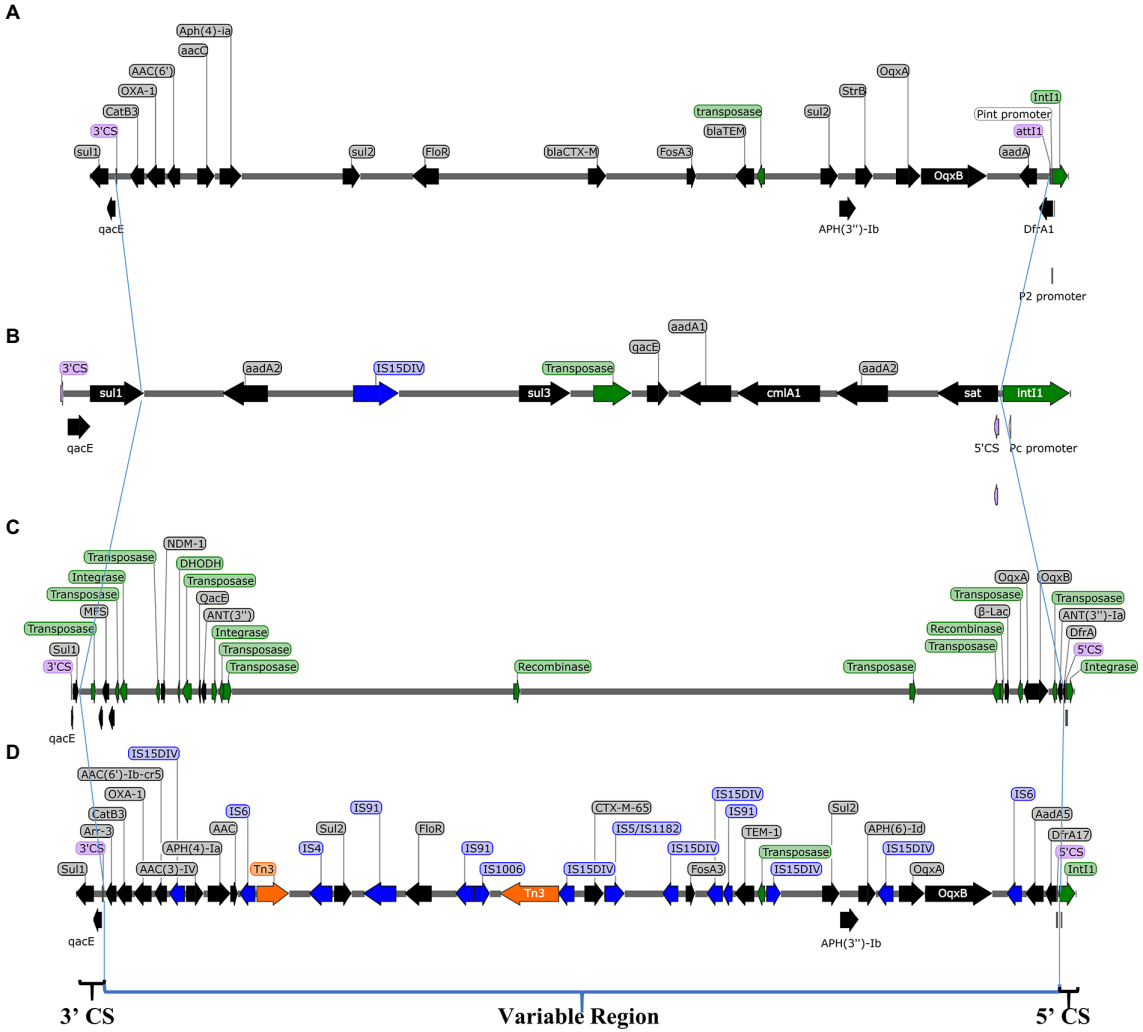
Structure of class 1 integron in plasmids of *S*. Indiana 15 **(A)**, *S*. Indiana 222 **(B)** isolates, *S*. Indiana C629 **(C)**, and *S*. Indiana D90 **(D)**. ARG, antibiotic resistance genes; IS, insertion sequence; Transposition protein, integrase, and recombinase.

*Salmonella* Indiana 15 and *S*. Indiana 222 isolates each contained a large plasmid; *S*. Indiana 15 contained 214,299 bp (accession number: CP092259) (Supplementary Figure 1), and *S*. Indiana 222 contained 308,622 bp (accession number: CP031190) (Supplementary Figure 2). The class 1 integron was located in the large plasmids of the *S*. Indiana 15 and *S*. Indiana 222 isolates (Supplementary Figures 1, 2), and the two ends were 5’CS and 3’CS, and the middle was a variable region (Figure 2). Twenty-two complete antimicrobial resistance genes were identified in the large plasmid of *S*. Indiana 222 (Table 2; Supplementary Figure 2), while the large plasmid of *S*. Indiana 15 had 19 complete antimicrobial resistance genes (Table 2; Supplementary Figure 1).

**Table 2:** Resistance genes of large plasmids of *S*. Indiana 15, *S*. Indiana 222, *S*. Indiana D90, and *S*. Indiana C629 isolates.

Antimicrobial resistance-encoding genes	*S*. Indiana 222-plasmid scf71810 (308,622 bp)	*S*. Indiana 15-plasmid1 (214,299 bp)	*S*. Indiana D90 pD90-1 (222,470 bp)	*S*. Indiana C629 pC629 (210,106 bp)
*aac6-Ib*	1	1	1	1
*aacC4/AAC(3)-IV*	1	1	1	1
*aadA1*	1			
*aadA2*	2			
*ANT(3″)*				2
*Aph(3″)-Ib*	1	1	1	
*Aph(4)-Ia*	1		1	
*Aph(6)-Id*	1	1	1	
*AphA1*	1	1		
*Arr-3*	1		1	1
*bla*OXA-1	1	1	1	1
*bla*TEM-1	1	1	1	1
*Ble*	1	1	1	1
*CatB3*	1	1	1	1
*bla*CTX-M		1	1	
*dfrA12*	1	1		1
*floR*	1	1	1	
*FosA3*			1	
*mphA*		4		2
*bla*NDM-1				1
*qacE*	2		1	2
*Sul1*	2	3	1	
*tet(A)*	1			
*tetR*	1			
Classes	19	14	15	12
Total number	22	19	15	15

### Comparison of the integration efficiency of two class 1 integrases *intI1*

Comparative analysis was conducted between the *intI1* (1,014 bp) of *S*. Indiana 222 and the *intI1* (699 bp) of *S*. Indiana 15. In terms of the gene size, *intI1* (699 bp) of *S*. Indiana 15 was 315 bp smaller than *intI1* (1,014 bp) of *S*. Indiana 222, and the gene sequence of former showed 67.26% coverage and 67.06% nucleotide identity with that of the latter gene. Analysis of the INTEGRALL database (see text footnote 1) found that the product encoded by *intI1* (1,014 bp) of *S*. Indiana 222 belongs to tyrosine recombinase XerD, while the product encoded by *intI1* (699 bp) of *S*. Indiana 15 belongs to tyrosine recombinase XerC.

**Figure 3:**
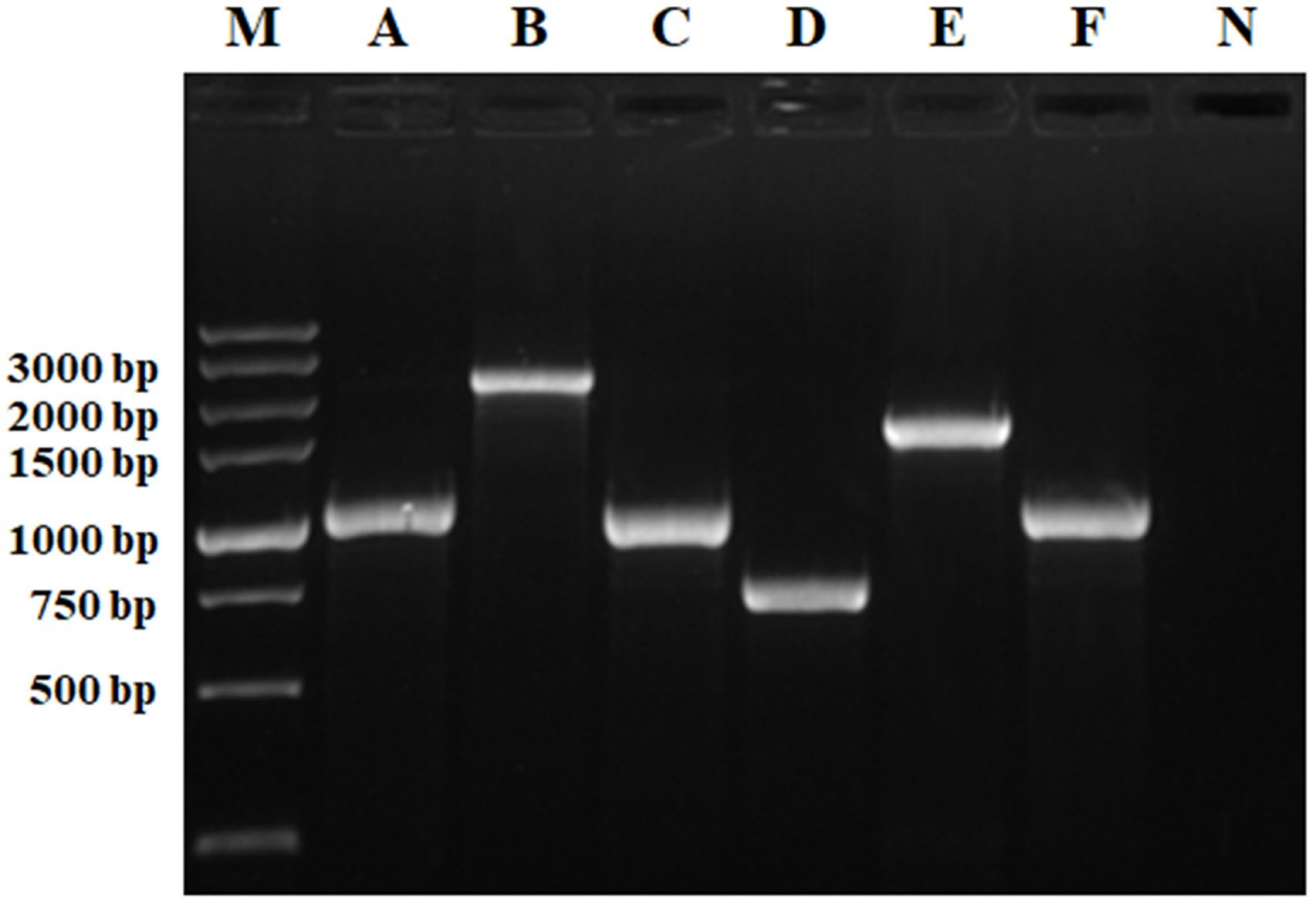
Identification of PCR amplification products. M, DL5000 DNA Marker; A, *intI1* (1,033 bp); B, INT (2,330 bp); C, *aadA2* (987 bp); D, *intI1-15* (717 bp); E, INT15 (1,526 bp); F, *aadA2-15* (985 bp); N, negative control.

The target genes were amplified by PCR using an existing *S*. Indiana 222 plasmid scf71810 and *S*. Indiana 15 plasmid1 as template. The electrophoresis result was consistent with the expected PCR amplicon size (Figure 3).

**Figure 4:**
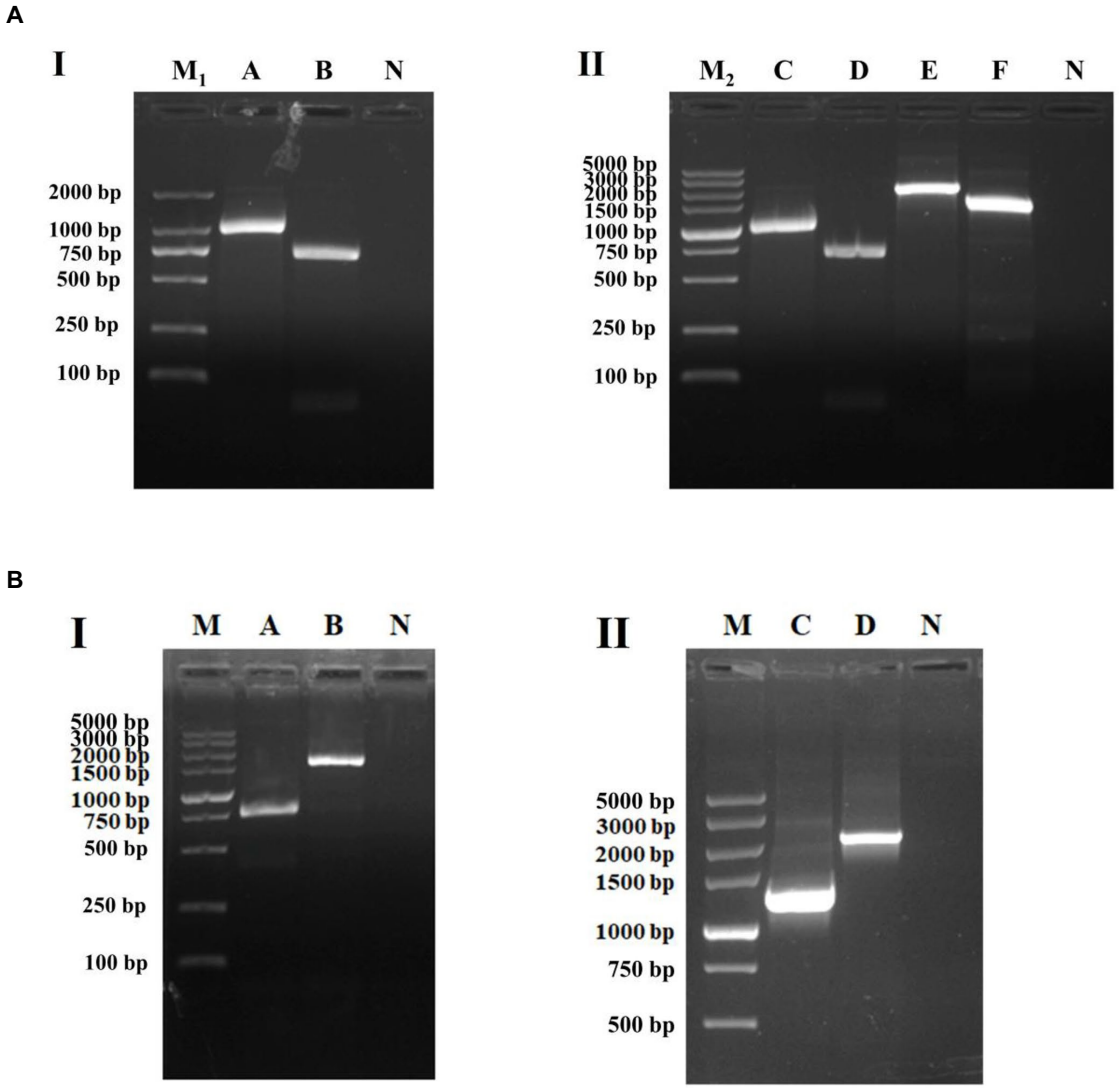
Identification of PCR amplification products **(A)** and integration **(B)**. I: Identification of pUCINTI1 and pUCINTI15 by PCR. M_1_, DL2000 DNA Marker; A, *intI1* (1,033 bp); B, *intI1-15* (717 bp); N, control; II, identification of pUCINTI1, pUCINTI15 and pACINT, pACINT15 by PCR. M_2_, DL5000 DNA Marker; C, *intI1* (1,033 bp); D, *intI1-15* (717 bp); E, INT (2,330 bp); F, INT15 (1,526 bp); N, control. I, identification of integration function of *intI1*. II, identification of integration function of *intI1-15*. M, DL5000 DNA Marker; A, before integrated (779 bp); B, after integrated (1,634 bp); C, before integrated (1,268 bp); D, after integrated (2,123 bp); N, control.

The purified PCR products, after sequenced correctly, were ligated into the pUC19 and pACYC184 Vector, respectively. After plasmids pUCINTI1, pACINT, and pUCINTI15, pACINT15 were transformed into *E*. coli BL21(DE3) competent cells by heat shock, in turn, they were detected with primers intI1-F/R, INT-F/R, intI15-F/R, and INT15-F/R, respectively. These results confirmed that the recombinant pUCINTI1, pUCINTI15, pACINT and pACINT15 vector were successfully constructed (Figure 4A).

After plasmids pACAAD and pACAAD15 were transformed into *E*. coli BL21(DE3) competent cells containing plasmids pUCINTI1, pACINT and pUCINTI15, pACINT15 by heat shock, respectively, we counted the number of bacterial clones on the LB agar plate and detected by polymerase chain reaction (PCR) to determine whether the *aadA2* gene was integrated. These results confirmed that both the *intI1* with the size of 1,014 bp and 699 bp integrated the *aadA2* gene cassette (Figure 4B).

**Figure 5:**
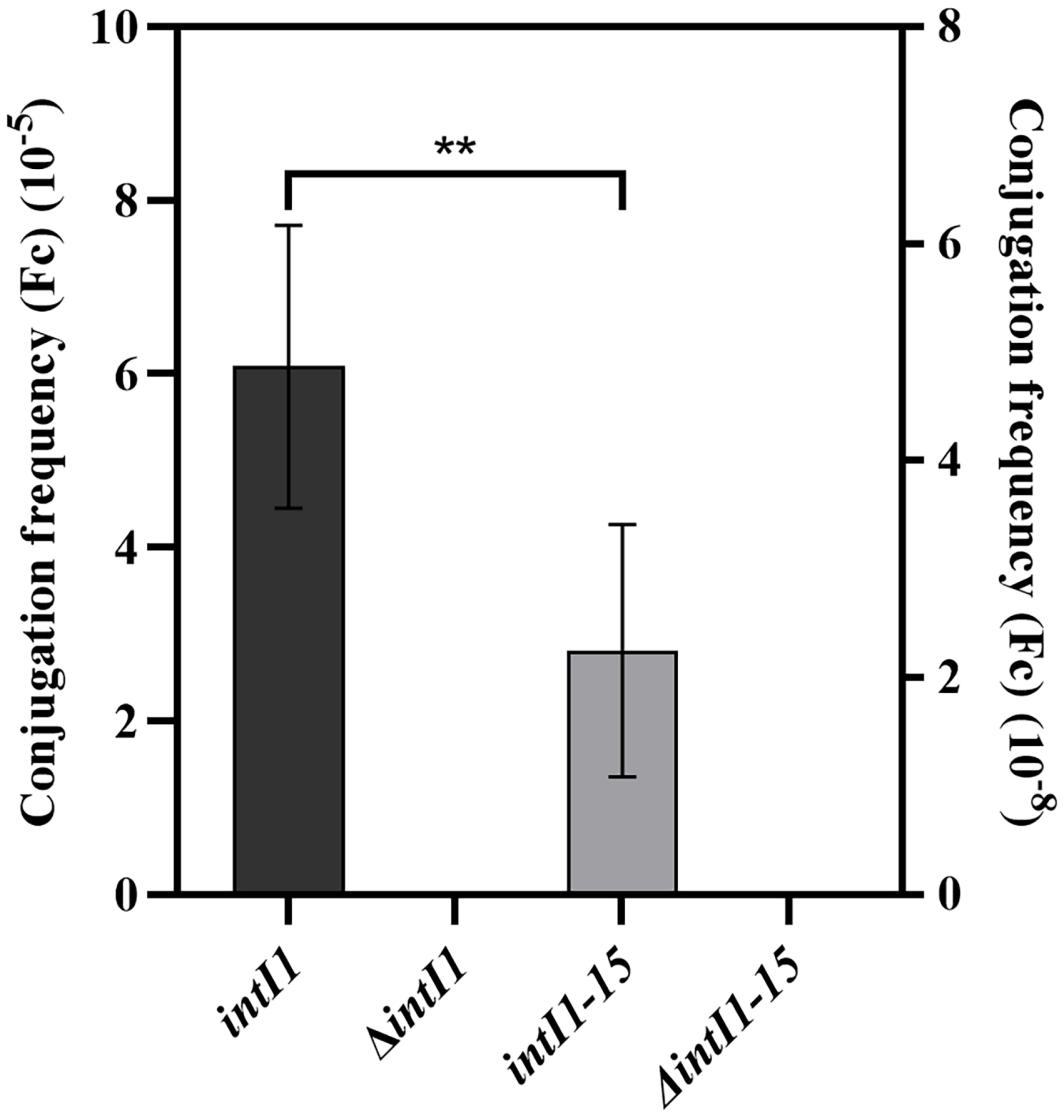
The conjugation frequency (Fc) of *intI1* and *intI1-15*. **p < 0.01.

The results of conjugation frequency (Fc) showed that the average conjugation frequency (Fc) of *intI1* (1,014 bp) was 6.08 × 10^−5^, while the average conjugation frequency (Fc) of *intI1* (699 bp) was 2.25 × 10^−8^. The conjugation frequency (Fc) of *intI1* (1,014 bp) was significantly higher than that of *intI1* (699 bp) (*p* < 0.01) (Figure 5).

### Correlations between class 1 integrons and the antibiotic resistance genes

**Figure 6:**
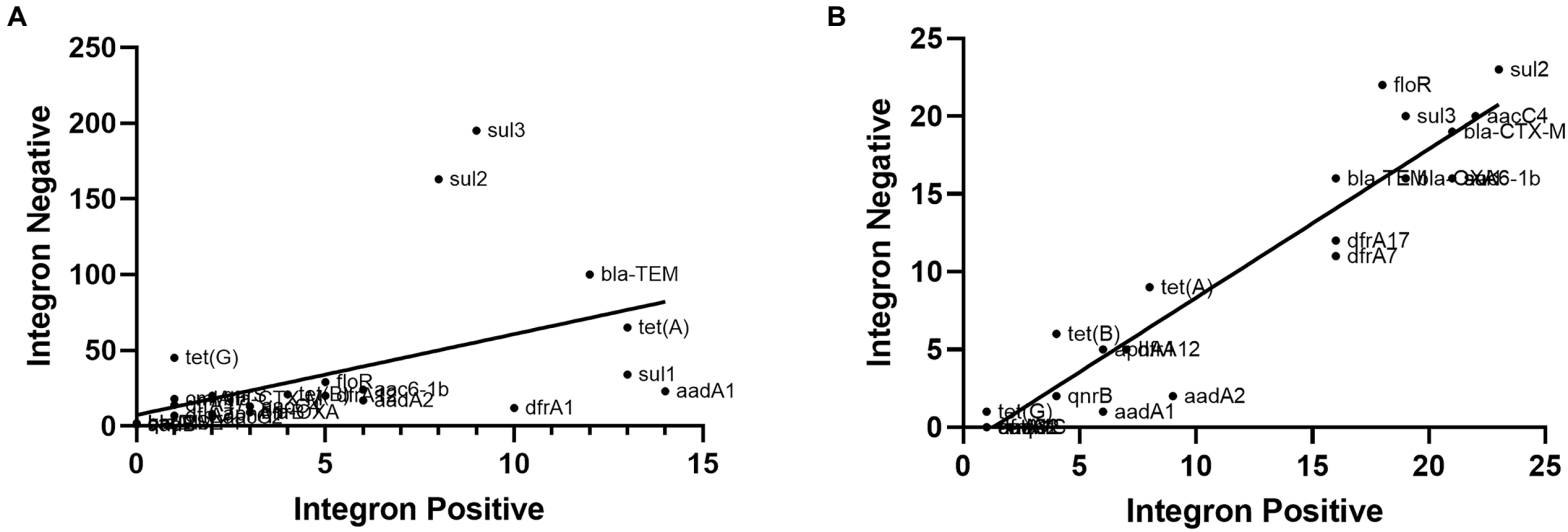
The correlations between class 1 integrons and antibiotic resistance genes of all 200 non-*Salmonella* Indiana isolates and *S*. Indiana isolates. **(A)** The correlations between class 1 integrons and antibiotic resistance genes of all 200 non-*Salmonella* Indiana isolates. **(B)** The correlations between class 1 integrons and antibiotic resistance genes of *S*. Indiana isolates.

Based on our previous study on resistance genes (Chao et al., 2017), we analyzed the correlations between class 1 integrons and the number of antibiotic resistance genes by calculating Pearson correlation coefficients. As illustrated in Figure 6, the class 1 integrons and the number of antibiotic resistance genes in *S*. Indiana isolates showed exceptionally strong correlations (*r* = 0.952, *p* < 0.001), while the class 1 integrons and the number of antibiotic resistance genes in all 200 non-*Salmonella* Indiana isolates showed a moderate correlation (*r* = 0.510, *p* < 0.001) (Figure 6). This indicated that *S*. Indiana may acquire a large number of resistance genes *via* class 1 integrons and integrate them into plasmids. We speculated that class 1 integrons could be one of the causes of the multidrug resistance of *S*. Indiana.

### Plasmid conjugation transfer assay

Two types of plasmids have been identified in *S*. Derby 146, *S*. Indiana 47, and *S*. Indiana 388: IncHI2 and IncN. IncHI2 plasmids were detected in *S*. Indiana 89, and no plasmids were detected in *S*. Enteritidis 147, *S*. Derby 368, and *S*. Enteritidis 242 (unpublished data).

The conjugation frequency of the integron-negative recipient isolate *S*. Indiana 388 was 9.36 × 10^−6^ (Table 3). The conjugation frequencies of the three integron-positive recipient isolates, *S*. Derby 146, *S*. Indiana 89, and *S*. Indiana 47, were 6.70 × 10^−5^, 3.44 × 10^−4^, and 3.62 × 10^−4^, respectively (Table 3). The conjugation frequencies of integron-positive *S*. Indiana recipient isolates were significantly higher than those of integron-negative *S*. Derby recipient isolates (*p* < 0.05).

**Table 3:** The conjugation frequency of four *Salmonella* isolates.

Donor	Resistant profile ^a^	Recipient	Resistant profile ^a^	Antibiotics (μg mL^−1^)	Resistance gene(s) transferred	Conjugation frequency	95% confident limit
*S*. Enteritidis 147 ^N, ND^	AMP, NA, TE	*S*. Derby 146 ^Y, AB^	AMP, CFP, NA, C	TE, 10; C, 25	*tetA*	6.70 × 10^−5^	10 ^(4.185 ± 0.115)^
*S*. Enteritidis 147 ^N, ND^	AMP, NA, TE	*S*. Indiana 388 ^N, AB^	CTX, CFP, K, CIP, C	TE, 10; C, 25	*tetA*	9.36 × 10^−6^	10 ^(5.032 ± 0.057)^
*S*. Derby 368 ^N, ND^	AMP, AMC, CTX, FOX, FEP, CFP, CRO, NA, SXT, TE	*S*. Indiana 89 ^P, A^	AMP, AMC, GN, K, NA, CIP, SXT, C	TE, 10; C, 25	*tetA*	3.44 × 10^−4^	10 ^(3.525 ± 0.239)^
*S*. Enteritidis 242 ^N, ND^	AMP, CTX, FEP, CFP, CRO, K, NA, C	*S*. Indiana 47 ^P, AB^	GN, K, AK, NA, CIP, SXT, C, TE	Amp, 20; TE, 10	*floR*, *tetB*	3.62 × 10^−4^	10 ^(3.555 ± 0.327)^

^N^, integron-negative; ^P^, integron-positive; ^A^, IncHI2; ^B^, IncN; ^ND^, not detected plasmids of any types.

a : AMP, ampicillin; AMC, amoxicillin-clavulanic acid; CTX, cefotaxime; FOX, cefoxitin; FEP, cefepime; CFP, cefoperazone; CRO, ceftriaxone; GN, gentamicin; K, kanamycin; AK, amikacin; NA, nalidixic acid; CIP, ciprofloxacin; SXT, trimethoprim/sulfamethoxazole; C, chloramphenicol; TE, tetracycline.

## Discussion

As important genetic elements in *Salmonella*, integrons can capture resistance genes and integrate them into their genome (Yang et al., 2009). Our results showed that most *S*. Indiana isolates carried class 1 integrons and the percentage of isolates carrying class 1 integrons significantly more than other *Salmonella* serotypes (*p* < 0.001). These values were much higher than the statistical results obtained in 2007 (Boucher et al., 2007). We speculated that this may be caused by the different sources and isolation environment and those studied by Boucher et al.

Some *S*. Indiana isolates were found to have MDR characteristics, demonstrating resistance to between 9 and 14 antibiotics and harboring a large number of drug resistance genes (Bai et al., 2015; Gong et al., 2016; Chao et al., 2017). We found that *S*. Indiana isolates carried plasmids and harbored class 1 integrons and multidrug resistance genes or gene clusters in their large plasmid, this indicated that the class 1 integrons located in large plasmids were one of the important causes of the presence of multidrug resistance genes or gene clusters. These results were similar to those of plasmids pD90-1 and pC629, which both harbored 15 complete resistance genes (Wang J. et al., 2017; Wang W. et al., 2017).

Integrons are one class of site-specific recombination elements which insert and excise mobile antibiotic resistance gene cassettes *via* integrase, and which are located on plasmids and/or transposons (Hall and Collis, 1995). Integron insert genes can also be excised by the integrase, and the circular gene cassette generated can be reinserted at a new location (Hall et al., 2010). *IntI1* integrase is a tyrosine recombinase involved in the mobility of antibiotic resistance gene cassettes within bacterial class 1 integrons (Dubois et al., 2007). Our results showed that compared with the *intI1* integrase of *S*. Indiana 222, the gene encoding integrase of *S*. Indiana 15 also belongs to tyrosine recombinase, and these two genes had higher coverage and nucleotide identity but were not aligned with other classes of integrase. We speculated that the integrase of *S*. Indiana 15 may be a mutant of *intI1* integrase.

Studies have shown that the integration frequency of *aadA2* gene cassette was 1.87 × 10^−4^ when *intI1* integrase was present (Yang et al., 2009). We used the same method to determine the integration frequency of these two integrases, and our work has shown that the average conjugation frequencies of *intI1* of *S*. Indiana 222 and *S*. Indiana 15 were 6.08 × 10^−5^ and 2.25 × 10^−8^, respectively. We speculated that the different integration frequency may be due to the different selection of integron gene. Sequence analysis showed that the *aadA2* gene cassette was integrated at the *attI* site of the integron, specifically at G↓TTAGAC (↓is the recombination site), which was consistent with the results of the previous studies (Bito and Susani, 1994; Rowe-Magnus and Mazel, 2001). Although the integration function of integrase in *S*. Indiana 15 isolate was significantly lower than that of *S*. Indiana 222 isolate (*p* < 0.01), this made *S*. Indiana strains had one more way to acquire resistance genes.

Studies have shown that the conjugation frequencies of bacteria with different types of plasmids were different (McDonnell et al., 1983; Collis et al., 2001). Our work has shown that the conjugation frequency of integron-positive *S*. Indiana recipient strains significantly exceeds that of the other recipient strains (*p* < 0.05), which explained why *S*. Indiana isolates containing integrons could more easily capture resistance genes from the other bacteria, hence causing the current pandemic of multidrug resistance clones.

In conclusion, the proportion of *S*. Indiana isolates carrying class1 integrons was significantly higher than those in other *Salmonella* serotypes, and a class 1 integron and a large number of resistance genes were harbored in large plasmids of *S*. Indiana isolates. Two integrases were found in *S*. Indiana isolates, which made *S*. Indiana strains had one more way to acquire resistance genes. The large plasmid carrying class 1 integrons mediated the multidrug resistance of *S*. Indiana. Our findings may shed light on the dissemination mechanism of resistance genes in *Salmonella* and, in turn, may have important implications for food safety and public health.

## Data availability statement

The datasets presented in this study can be found in online repositories. The names of the repository/repositories and accession number(s) can be found in the article/Supplementary material.

## Author contributions

GC designed the manuscript. XW and GC wrote the manuscript. All authors took full responsibility for the study design, data analysis and interpretation, and preparation of the manuscript. All authors contributed to the article and approved the submitted version.

## Funding

This work was supported by the National Key Research and Development Project of China (2016YFD0501609), Health Innovation Team of Yangzhou (LJRC201826), Fund of Jiangsu Dairy Biotechnology Research Center (KYRY2019010), the Earmarked Fund for Modern Agroindustry Technology Research System (CARS-40-K16), The “High-end Talent Support Program” of Yangzhou University (2016), and a project funded by the Priority Academic Program Development of Jiangsu Higher Education Institutions (PAPD).

## Conflict of interest

The authors declare that the research was conducted in the absence of any commercial or financial relationships that could be construed as a potential conflict of interest.

## Publisher’s Note

All claims expressed in this article are solely those of the authors and do not necessarily represent those of their affiliated organizations, or those of the publisher, the editors and the reviewers. Any product that may be evaluated in this article, or claim that may be made by its manufacturer, is not guaranteed or endorsed by the publisher.

## Footnotes

^1^ http://integrall.bio.ua.pt/

^2^ https://blast.ncbi.nlm.nih.gov/Blast.cgi

^3^ http://www.bcgsc.ca/platform/bioinfo/software/abyss

^4^ https://github.com/marbl/canu

^5^ https://sourceforge.net/projects/soapdenovo2/files/GapCloser/

^6^ https://card.mcmaster.ca/

^7^ http://www.ncbi.nlm.nih.gov/orffinder
