# Supplementary material for: The large plasmid carried class 1 integrons mediated multidrug resistance of foodborne Salmonella Indiana
Source: Front Microbiol. 2022 Oct 14;13:991326. doi: 10.3389/fmicb.2022.991326 (PMC9614373; doi:10.3389/fmicb.2022.991326)
Supplement: Supplementary file 1 [file Data_Sheet_1.DOCX]

**
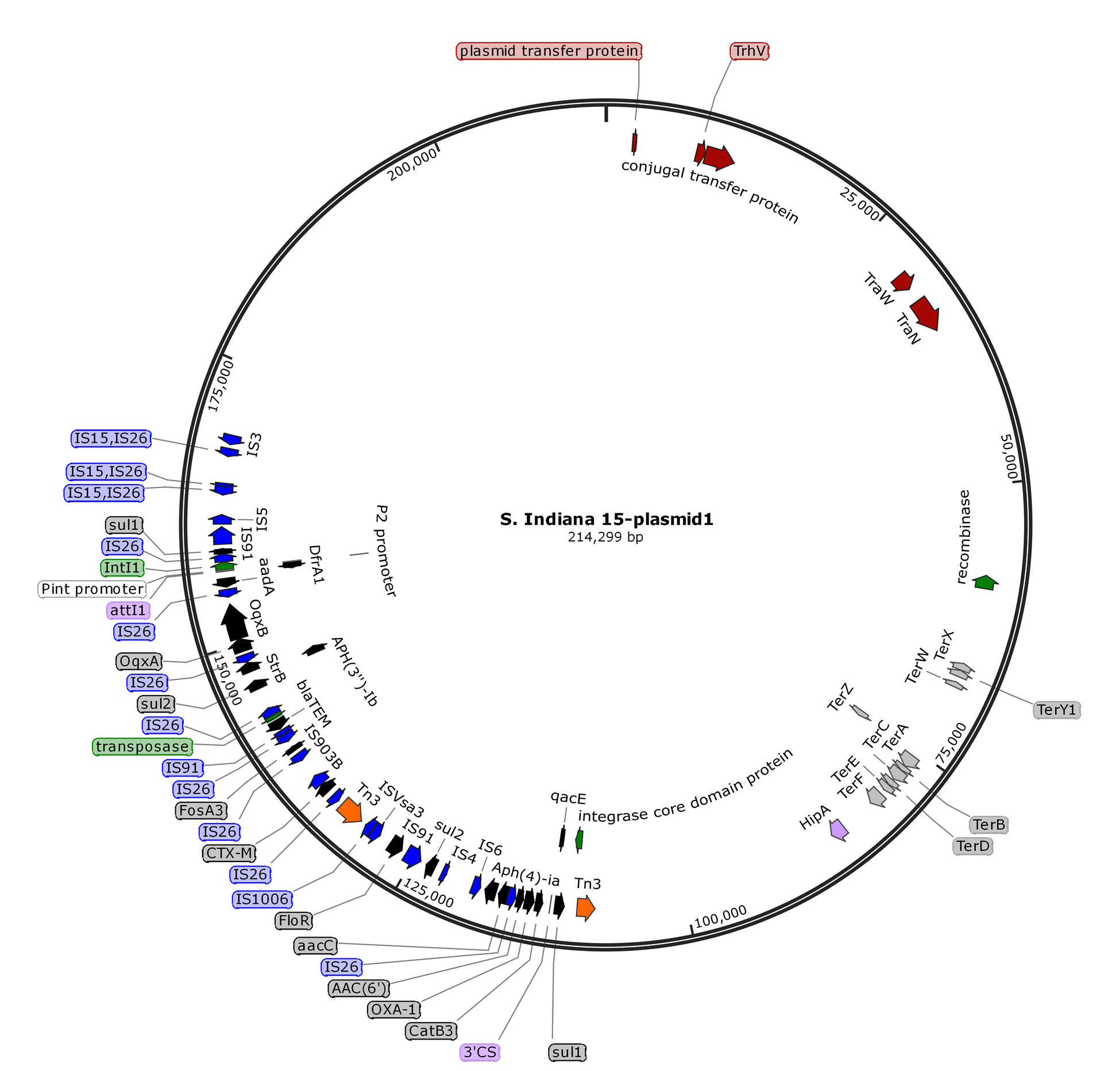
**

**Supplementary Figure 1**. The plasmid map of *S*. Indiana 15-plasmid1.

Antibiotic resistance genes (ARG); Insertion sequence (IS); Metal resistance genes (MRG); Transposition protein; Plasmid conjugative transfer protein; Integrase and recombinase; Toxin related protein.


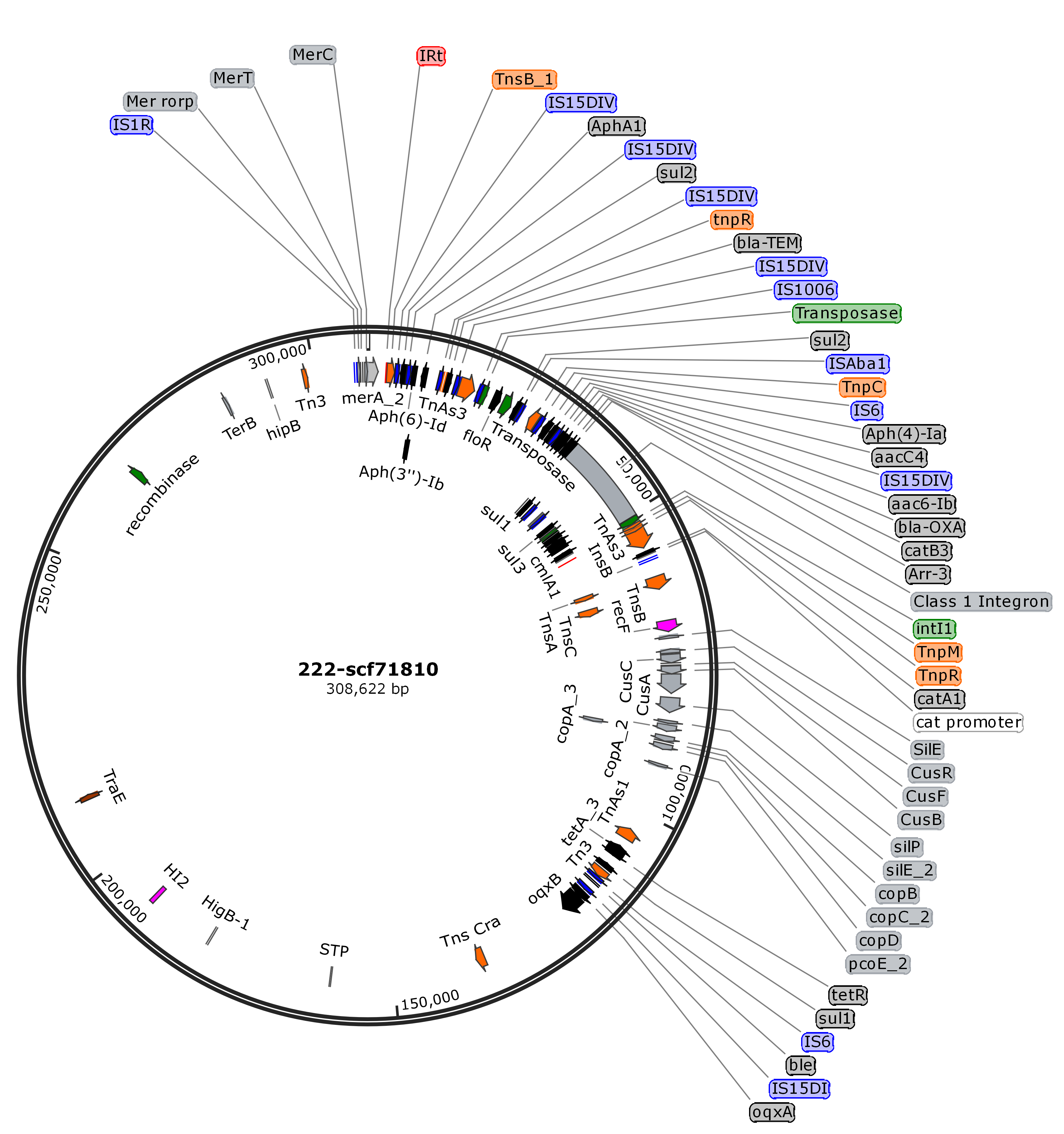


**Supplementary Figure 2**. The plasmid map of *S*. Indiana 222 scf71810 (accession number: CP031190).

Antibiotic resistance genes (ARG); Insertion sequence (IS); Metal resistance genes (MRG); Transposition protein; Plasmid conjugative transfer protein; Integrase and recombinase; Toxin related protein.
